# A Randomized Thorough QT Study of Apomorphine Sublingual Film in Patients With Parkinson's Disease

**DOI:** 10.1002/cpdd.1147

**Published:** 2022-07-28

**Authors:** Fabrizio Stocchi, Elizabeth L. Peckham, Maria Francesca De Pandis, Ken Sciarappa, Robert Kleiman, Felix Agbo, C. Warren Olanow, David Blum, Bradford Navia

**Affiliations:** ^1^ University San Raffaele and Institute for Research and Medical Care, IRCCS San Raffaele Pisana Rome Italy; ^2^ Central Texas Neurology Consultants Round Rock Texas USA; ^3^ San Raffaele Cassino Tosinvest Sanità Cassino Italy; ^4^ Sunovion Pharmaceuticals Inc. Marlborough Massachusetts USA; ^5^ eResearch Technology Inc. Philadelphia Pennsylvania USA; ^6^ Sunovion Pharmaceuticals Inc. Teaneck New Jersey USA; ^7^ Mount Sinai School of Medicine New York New York USA; ^8^ Clintrex Sarasota Florida USA

**Keywords:** apomorphine sublingual film, “OFF” episodes, Parkinson's disease, pharmacokinetics, QT interval

## Abstract

A randomized thorough QT study was conducted to assess the effects of apomorphine sublingual film (SL‐APO) on corrected QT interval (QTc) and other cardiac conduction parameters in patients with Parkinson's disease (PD) and “OFF” episodes. Patients were titrated to an SL‐APO dose that resulted in FULL “ON,” followed by up to two additional doses (maximum 60 mg), then randomized at the highest tolerated dose to a treatment sequence of SL‐APO, placebo, and moxifloxacin (400 mg, positive control) in a three‐way crossover design. Changes from baseline in time‐matched, placebo‐adjusted Fridericia‐corrected QTc interval (ΔΔQTcF) and Bazett‐corrected QTc interval (ΔΔQTcB) were analyzed from postdose electrocardiograms. Forty patients were randomized and received single doses of study treatments. Upper limits of 90% confidence intervals (CIs) for ΔΔQTcF of SL‐APO were below the 10‐millisecond regulatory threshold at all prespecified timepoints, demonstrating no clinically significant effect on QTcF. Lower limits of 90% CIs for ΔΔQTcF of moxifloxacin exceeded the 5‐millisecond regulatory threshold at all timepoints up to 3 hours, confirming assay sensitivity. SL‐APO had no clinically meaningful effects on QTcB, PR/QRS intervals, heart rate, or electrocardiogram‐derived morphology (EudraCT identifier: 2016‐001762‐29; ClinicalTrials.gov identifier: NCT03187301).

Parkinson's disease (PD) is a chronic and progressive disorder affecting approximately 1 million people in the United States alone.^1^, ^2^ Levodopa is the gold standard for the treatment of PD, but chronic levodopa treatment is associated with the development of motor fluctuations in a majority of patients.^3^ As a consequence of levodopa pharmacokinetics, patients experience motor fluctuations consisting of increasingly shorter periods of symptom improvement (“ON” time), often associated with dyskinesia (involuntary movements most often associated with peak levodopa concentrations), and increasing time when PD motor and nonmotor symptoms resurface (“OFF” episodes).^4^, ^5^ As a result of PD progression, patients need adjustments to their initial therapy accompanied by additional treatments that increase “ON” time (ie, “ON‐extenders”) or acute, intermittent treatments to manage “OFF” episodes, along with therapies to reduce dyskinesia.^1^, ^6^

Patients with PD may be predisposed to a prolonged QT interval and are at risk of developing other cardiac abnormalities compared with healthy aged‐matched individuals.^7^, ^8^ The tendency towards a prolonged QT interval in some patients is likely attributable to autonomic cardiac dysfunction resulting from neuronal degeneration.^9^ Risk factors include advanced PD and confounding medications used in PD management, such as domperidone, amantadine, and antidepressants including citalopram and amitriptyline.^10^, ^11^

Apomorphine, a potent, non‐ergoline dopamine agonist with anti‐parkinsonian benefits comparable to oral carbidopa/levodopa, is used as an acute, intermittent treatment for individual “OFF” episodes in patients with PD.^12^, ^13^, ^14^, ^15^ Prolongation of the QT interval has been reported in the literature and approved product labeling for subcutaneous apomorphine at exposure to the higher end of the recommended dose range (2‒6 mg).^16^ A significant exposure–response relationship was also identified between subcutaneously administered apomorphine concentration and Fredericia‐corrected QTc interval (QTcF), as described in the approved product labeling.

Apomorphine sublingual film, a novel sublingual formulation of apomorphine, is approved for the acute, intermittent treatment of “OFF” episodes associated with PD.^17^ A prior study found that maximum plasma concentration (C_max_) was lower and time to C_max_ (T_max_) was longer for apomorphine sublingual film compared with subcutaneous apomorphine formulations, while overall systemic apomorphine exposure was similar.^18^ The pharmacokinetic properties of apomorphine sublingual film may contribute to a more favorable safety profile than other apomorphine formulations, as similar overall exposure is achieved without a rapid early rise in drug concentration. In a pivotal study, apomorphine sublingual film demonstrated a significant improvement in the Movement Disorder Society Unified Parkinson's Disease Rating Scale Part III motor examination score at 30 minutes postdose compared with placebo (least squares [LS] mean difference ‒7.6, *P *= 0.0002).^19^ Apomorphine sublingual film was generally safe and well tolerated; the most common (≥10%) treatment‐emergent adverse events (TEAEs) were nausea (21%), yawning (12%), dizziness (11%), and somnolence (11%) during the open‐label titration phase and nausea (28%) and somnolence (13%) during the 12‐week double‐blind maintenance phase.^19^ The current study evaluated the effects of apomorphine sublingual film on the corrected QT interval (QTc) and other cardiac conduction parameters in patients with PD and “OFF” episodes.

## Methods

### Patients

The study enrolled adult patients with idiopathic PD, consistent with UK Brain Bank criteria, who were responsive to oral carbidopa/levodopa and able to demonstrate a drug‐withdrawal–induced “OFF” episode. Patients who were receiving stable doses of anti‐parkinsonian medication (oral carbidopa/levodopa and adjunctive PD medications), with a clear difference in “OFF” and “ON” states as determined by the investigator, were eligible to participate. Exclusion criteria included atypical or secondary parkinsonism, history of dopamine agonist–related nausea that required the use of antiemetics, neurosurgical treatment for PD, use of apomorphine infusion or carbidopa/levodopa intestinal infusion, contraindications for moxifloxacin or apomorphine or sensitivity to apomorphine, or clinically significant cardiac and/or electrocardiogram (ECG) abnormalities at screening. A protocol amendment expanded the eligibility criteria to increase enrollment by allowing inclusion of patients with less severe disease (eg, patients experiencing “OFF” episodes but without consistent, well‐defined “OFF” episodes, and patients receiving levodopa at least three times per day instead of at least four times per day were subsequently allowed) and patients previously enrolled in a phase 3 study of apomorphine sublingual film.

### Study Design

This randomized, double‐blind, placebo‐controlled, three‐way crossover study was conducted at 13 clinical sites in Italy and the United States. The study was initially registered in November 2016 (EudraCT identifier: 2016‐001762‐29; ClinicalTrials.gov identifier: NCT03187301) and was conducted from April to December 2017. It was determined that healthy volunteers would be susceptible to the emetic effects of apomorphine, particularly at higher doses, and patients with PD were therefore deemed a more suitable study population. Based on investigator discretion, patients entered the open‐label dose‐titration phase in a practically defined “OFF” (ie, PD medication withheld after midnight the night before) or when “OFF” after they took their normal morning dose of PD medication but before taking their next dose of medication. Titration began once the patient was confirmed to be “OFF” by both the investigator and the patient. The starting dose of apomorphine sublingual film was 10 mg and doses were increased in 5‐mg increments up to 40 mg and then in 10‐mg increments up to 60 mg, as tolerated, until a FULL “ON” was achieved within 90 minutes postdose and confirmed by patient and investigator (Figure S1). FULL “ON” was defined as a response comparable to that with oral carbidopa/levodopa. Next, apomorphine sublingual film dose was further increased as tolerated up to two dose levels higher to a maximum of 60 mg (eg, if FULL “ON” was achieved at 10 mg, dosing continued until 20 mg if tolerated).^20^ Patients who were unable to tolerate the additional dose after achieving a FULL “ON” were allocated to receive treatment at the previous dose level. Use of antiemetics, including trimethobenzamide (US) and domperidone (Italy), was not allowed during the study.

Patients who successfully completed the open‐label titration phase entered the crossover phase, where they were randomized using a three‐way balanced crossover design to a single dose of apomorphine sublingual film, administered in a blinded fashion at the highest tolerated dose determined during titration, matching placebo (identical in size, shape, color, and appearance) administered in a blinded fashion and moxifloxacin (400 mg) administered open‐label (Figure S1). After a 2‐ to 7‐day washout between the final dose‐titration phase visit and Period 1 of the crossover, patients were randomized to six possible treatment sequences using central randomization with no stratification, and a 3‐day washout period occurred between treatments. The randomization scheme was designed by the study statistician, and an independent expert executed the randomization via an interactive web response system. Patients and staff were blinded to treatment assignments until study completion.

The study was approved by a central institutional review board (IRB) (Copernicus Group [Research Triangle Park, North Carolina] or IRCCS San Raffaele Pisana Ethics Committee [Rome, Italy]) or, if required, by the local IRB for a given site (independent ethics committee at PTV Polyclinic Tor Vergata Foundation [Rome, Italy], Lazio 2 Ethics Committee [Rome, Italy], Ethics Committee of the Provinces of Chieti and Pescara [Chieti, Italy], and SUNY Downstate Medical Center [Brooklyn, New York]). The study was conducted in accordance with the International Conference on Harmonization Guideline for Good Clinical Practice and the Declaration of Helsinki. Written informed consent was obtained from all patients before any study activity or procedure was undertaken.

### Assessments

Demographics, baseline characteristics, and medical history were recorded for all patients. Resting 12‐lead ECGs were performed at every study visit, and ambulatory ECGs were performed at each visit during the crossover phase. Three sets of triplicate ECGs were recorded at baseline and a single triplicate at 0.25, 0.5, 0.75, 1, 2, 3, 4, 8, 12, and 24 hours postdose. Blood samples for pharmacokinetic analysis were collected at each visit, with plasma apomorphine concentrations assessed predose and at 0.5, 0.75, 1, 2, and 4 hours postdose for apomorphine sublingual film and placebo and at 0.5, 1, 2, 3, 4, 6, and 8 hours postdose for moxifloxacin. Vital signs (blood pressure, heart rate [HR], respiratory rate, and body temperature) and TEAEs were monitored throughout the study.

### Analytic Procedure

The methodology for the quantification of apomorphine has been previously published.^21^ Briefly, plasma concentrations of apomorphine and its metabolite were measured using a validated liquid chromatography‐tandem mass spectrometry method. The methodology for quantification of moxifloxacin has also been previously published.^22^, ^23^ Briefly, high‐performance liquid chromatography was used to measure the plasma concentration of moxifloxacin.

### Statistical Analysis

The safety population included all patients who received ≥1 dose of apomorphine sublingual film and the crossover population included all patients who received ≥1 dose of study drug after randomization. The ECG population, used for primary and related secondary analyses, included all patients who had a baseline ambulatory ECG and ≥1 postdose ECG after randomization. The pharmacokinetics population included all patients who had ≥1 pharmacokinetic evaluation.

The study was powered to detect a mean difference of 7 milliseconds for change in QTcF between apomorphine sublingual film and placebo (ΔΔQTcF), assuming the true difference could be up to 3 milliseconds and the standard deviation was 14 milliseconds.^24^ Assuming a one‐sided significance level of 0.05, 42 patients were required to achieve approximately 80% power with adjustment for inverse multiplicity. The primary analysis, assessed in the ECG population, was change from baseline in QTc interval, which was time‐matched, placebo‐adjusted, and corrected for HR using ΔΔQTcF. Changes from baseline were compared between apomorphine sublingual film and placebo at 0.25, 0.5, 0.75, 1, 2, 3, and 4 hours postdose. Baseline was defined as the mean of the nine ECGs recorded before dosing. If the upper limit of the two‐sided 90% confidence intervals (CIs) was <10 milliseconds, it was concluded that apomorphine sublingual film did not prolong the QTc interval to a clinically significant degree. The analysis of QTcF was performed using a mixed model for repeated measures (MMRM) appropriate for the crossover design. The MMRM had the observed QTcF changes from baseline (ΔQTcF) at 0.25, 0.5, 0.75, 1, 2, 3, and 4 hours postdose as the response values, with region (Italy/US), sex, sequence (ABC, ACB, BCA, BAC, CAB, or CBA), period (1, 2, or 3), treatment (apomorphine sublingual film, placebo, or moxifloxacin), time (0.25, 0.5, 0.75, 1, 2, 3, and 4 hours) and interaction between treatment and time as fixed factors and baseline QTcF as a covariate. The patient nested within the sequence was included as a random effect, and a spatial power covariance structure was used for unequally spaced repeated measures over time.^25^ Degrees of freedom were computed using the Kenward–Roger method.^26^

Assay sensitivity was evaluated with moxifloxacin, which is known to prolong the QT interval.^27^ This analysis used a similar statistical approach as the primary analysis, with the differences between moxifloxacin and placebo assessed at 1, 2, 3, and 4 hours postdose. Bonferroni‐corrected 90% CIs were calculated using a two‐sided coverage of 0.10/4 = 0.025 for the four CIs. If the lower limit of the one‐sided Bonferroni‐corrected 95% CIs was ≥5 milliseconds (threshold)^27^ at any prespecified timepoint, adequate sensitivity to assess QTc prolongation was demonstrated. A supportive, time‐averaged analysis was performed in which the mean of all baseline QTcF values was subtracted from the mean of all postdose QTcF values through 4 hours postdose, and the difference between apomorphine sublingual film and placebo was estimated. An outlier analysis was also conducted to reveal any additional effects on ECG intervals that would not have been detected in the primary analysis.

Secondary analyses included change from baseline in the Bazett‐corrected QTc interval (QTcB), HR, PR interval, QRS interval, and the uncorrected QT interval, which were assessed using an MMRM approach. Other secondary analyses included ECG morphology and the presence of cardiac arrhythmias, which were tabulated by treatment group. Pharmacokinetic parameters, including C_max_, T_max_, and area under the concentration–time curve from the time of dosing to the last measurable concentration (AUC_last_), were summarized with descriptive statistics for each dose level and were derived using noncompartmental methods employing Phoenix WinNonlin, version 6.3 (Certara, Princeton, New Jersey). Dose proportionality was investigated using a power model. Safety assessments included 12‐lead ECGs (resting), TEAEs, physical examination (including the oropharyngeal cavity), and vital signs (including orthostatic hypotension).

## Results

### Patients

**Figure 1:**
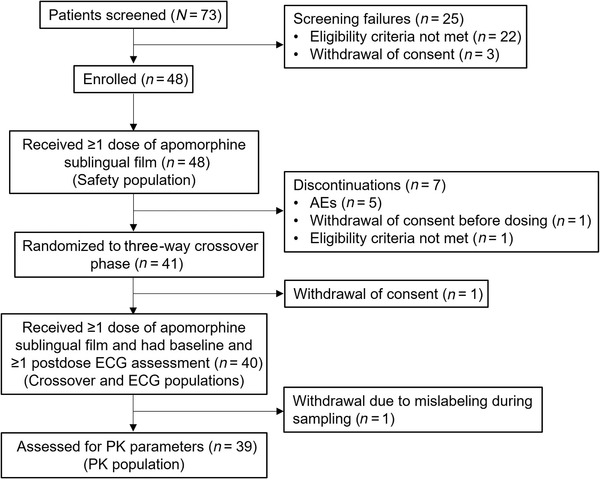
Patient disposition. AE, adverse event; ECG, electrocardiogram, PK, pharmacokinetics.

A total of 73 patients were screened, 48 (65.8%) of whom were enrolled in the open‐label titration phase, received at least one dose of apomorphine sublingual film, and were included in the safety population (Figure 1). Of these, seven patients discontinued as a result of adverse events (n = 5 [10.4%]; nausea, vomiting, nausea and vomiting, somnolence, and orthostatic hypotension), withdrawal of consent (n = 1 [2.1%]), and not meeting eligibility criteria (n = 1 [2.1%]; intracranial aneurysm identified after enrollment but before randomization). One patient withdrew consent after randomization but before receiving treatment; therefore, 40 patients were randomized to the crossover phase and included in the crossover and ECG analysis populations (Figure 1). Blood samples were obtained from 39 patients for pharmacokinetic analysis. Baseline demographics and clinical characteristics were similar across populations; most patients in the safety and crossover populations, respectively, were male (62.5% and 65.0%), White (91.7% and 92.5%), had a mean PD duration of 8.5 and 8.3 years, had motor fluctuations for a mean of 5.1 years, and received mean total daily levodopa doses of 632.7 and 620.5 mg (Table 1).

**Table 1:** Patient Demographics and Clinical Characteristics at Baseline

Characteristic	Safety Population (N = 48)	Crossover Population (N = 40)
Age, mean (SD), y	64.9 (8.56)	63.7 (8.68)
Male, n (%)	30 (62.5)	26 (65.0)
Race, n (%)		
White	44 (91.7)	37 (92.5)
Black	4 (8.3)	3 (7.5)
Time since PD diagnosis, mean (SD), y	8.5 (4.36)	8.3 (4.32)
Time since motor fluctuations started, mean (SD),^a^ y	5.1 (4.04)	5.1 (3.81)
Time since levodopa initiation, mean (SD), y	6.2 (4.35)	6.2 (4.5)
Total daily levodopa dose, mean (SD), mg	632.7 (281.54)	620.5 (273.05)

PD, Parkinson's disease; SD, standard deviation.

^a^ Data were missing for one patient: n = 47 (safety population) and n = 39 (crossover population).

### Apomorphine Sublingual Film Exposure

Among 48 patients who underwent dose titration, 68.3% of patients achieved a FULL “ON” with 10 mg of apomorphine sublingual film. All patients achieved FULL “ON” with doses ≤35 mg. After administration of up to two dose levels higher than the dose initially needed to achieve FULL “ON,” the highest dose received during titration was ≤20 mg for 79.2% of patients (10 mg, n = 9 [18.8%]; 15 mg, n = 11 [22.9%]; 20 mg, n = 18 [37.5%]; 25 mg, n = 5 [10.4%]; 35 mg, n = 1 [2.1%]; 40 mg, n = 2 [4.2%]; 50 mg, n = 2 [4.2%]); no patient had their dose titrated to 60 mg. Among 41 patients randomized to the crossover phase and dosed, 90.2% of patients received apomorphine sublingual film at a dose of <35 mg (10 mg, n = 16 [39.0%, includes one patient who discontinued before dosing]; 15 mg, n = 4 [9.8%]; 20 mg, n = 15 [36.6%]; 25 mg, n = 2 [4.9%]; 35 mg, n = 3 [7.3%]; 50 mg, n = 1 [2.4%]). Twenty patients (48.8%) were randomized to receive a lower dose than the highest dose received during titration due to tolerability (Table S1).

### Assessment of QT Interval Prolongation: Crossover Population

**Figure 2:**
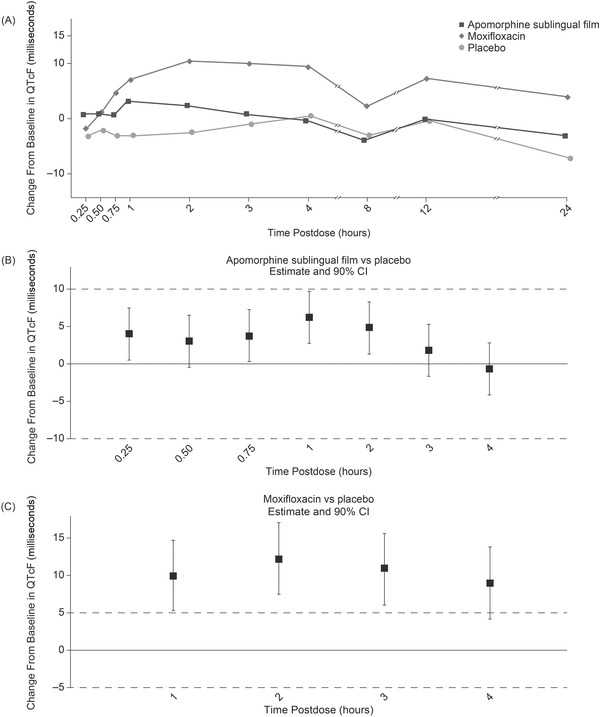
Mean changes from baseline in QTcF with (A) apomorphine sublingual film/placebo/moxifloxacin, (B) apomorphine sublingual film (time‐matched/placebo‐adjusted), and (C) moxifloxacin (time‐matched/placebo‐adjusted). CI, confidence interval; QTcF, Fridericia‐corrected QTc interval.

Administration of apomorphine sublingual film resulted in a mean change from baseline in QTcF that increased by up to 3.3 milliseconds at 1 hour postdose and was negative by 4 hours postdose (Figure 2A). Plasma moxifloxacin was measurable from 0.5 to 8 hours postdose with a mean peak of 1920 ng/mL at 2 hours postdose, and assay sensitivity was confirmed by a prolonged QT interval after moxifloxacin exposure in which QTcF increased up to 10.4 milliseconds by 2 hours postdose and then plateaued until 4 hours postdose before decreasing (Figures S2 and S3). As expected, placebo did not increase QTcF. The upper limits of the 90% CIs for ΔΔQTcF were below the regulatory threshold (10 milliseconds) at all timepoints, demonstrating that apomorphine sublingual film did not have a significant effect on QTcF (Figure 2B). Peak effect occurred at 1 hour postdose (6.2 milliseconds, 90% CI 2.7‒9.7). The lower limits of the 90% CIs for moxifloxacin versus placebo exceeded the regulatory threshold (5 milliseconds) at all prespecified timepoints up to 3 hours, confirming assay sensitivity (Figure 2C). The results of the primary analysis were consistent with the time‐averaged mean difference of 3.2 milliseconds (90% CI 1.1‒5.4) between apomorphine sublingual film and placebo. A summary of the change from baseline in QTcF by timepoint and treatment is provided in Table S2, and a scatterplot of RR interval versus QTcF is provided in Figure S4.

### Additional ECG‐Related Assessments

QTcB results were consistent with those of QTcF at all prespecified timepoints, with all upper limits of the 90% CIs below the threshold (10 milliseconds). Peak effect was seen at 2 hours postdose (5.0 milliseconds, 90% CI 0.8‒9.2). The uncorrected QT analysis showed similar findings to the QTcF analysis. The maximum LS mean change from baseline in the PR interval was 60 minutes postdose (2.5 milliseconds), and overall PR findings were similar to those with placebo.

No differences were observed in the incidence of postdose ECG abnormalities after administration of apomorphine sublingual film (15 patients [37.5%]), placebo (13 patients [32.5%]), and moxifloxacin (12 patients [30.0%]). The incidence of conduction abnormalities was similar after administration of apomorphine sublingual film (20 patients [50.0%]) and placebo (21 patients [52.5%]), with fewer incidences observed with moxifloxacin (13 patients [32.5%]). The most clinically relevant new postdose findings included first‐degree atrioventricular block in two patients, which was observed after apomorphine sublingual film, moxifloxacin, and placebo administration in one patient and after apomorphine sublingual film and placebo administration in the other. Atrial fibrillation occurred in one patient after apomorphine sublingual film administration, junctional rhythm in one patient after both apomorphine sublingual film and placebo administration, and nodal arrhythmia in one patient after apomorphine sublingual film administration (reported as a TEAE unrelated to study drug).

The outlier analysis revealed that no patients had a QTcF or QTcB >500 milliseconds in any of the treatment groups (Table S3). Overall, the number of QTcF outliers at any single timepoint was low (moxifloxacin ≤7 patients, apomorphine sublingual film ≤3, placebo ≤3). The number of QTcB outliers was higher (moxifloxacin ≤11 patients, apomorphine sublingual film ≤10, placebo ≤7), but the distribution was comparable with that of QTcF. Only a few HR, QRS, and QT interval outliers were observed, no PR interval outliers were found, and no difference between treatment groups was observed (Table S3). Summary data of the change from baseline in QRS interval, HR, and PR interval by timepoint and treatment are provided in Tables S4‒S6.

### Pharmacokinetics

**Figure 3:**
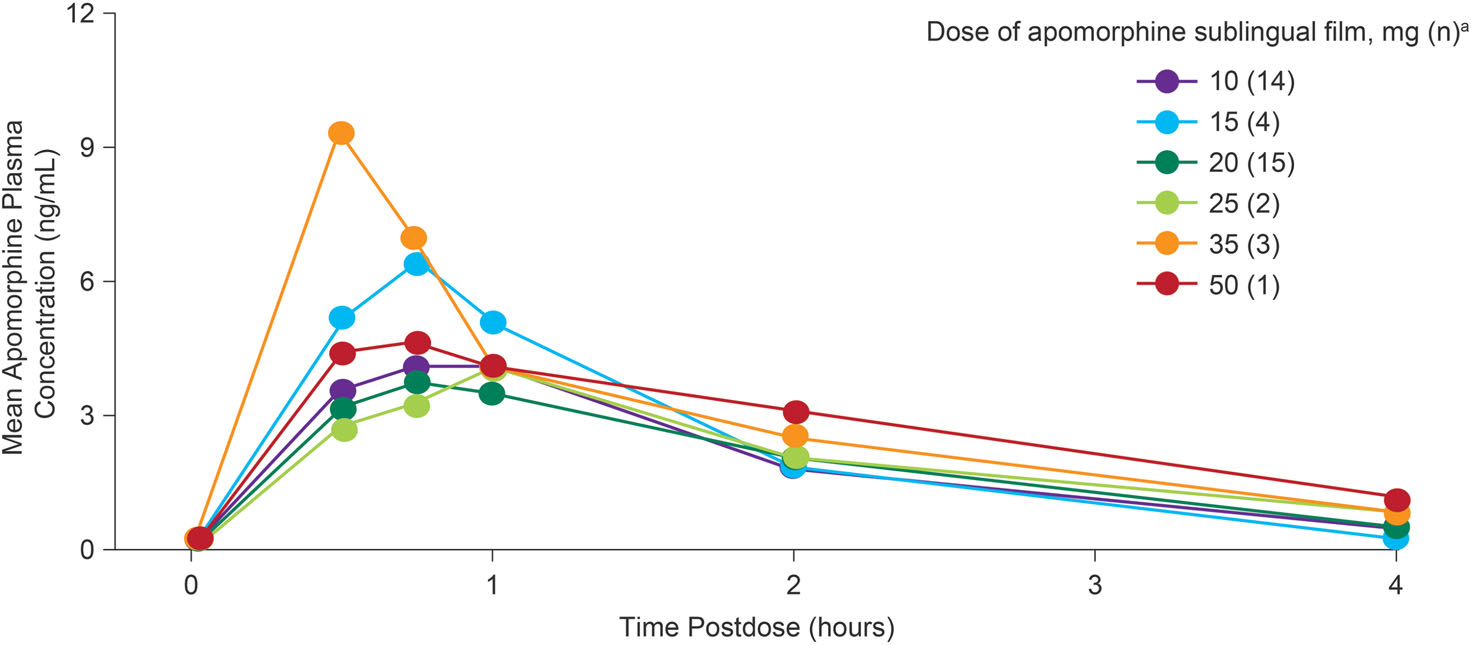
Mean apomorphine plasma concentration–time profiles by dose. ^a^Number of patients who received the indicated dose of apomorphine sublingual film.

Apomorphine plasma concentrations were measurable over the sample collection time (0.5‒4 hours postdose). Median T_max_ for apomorphine across all dose levels ranged from 0.58 to 1.50 hours. Across the 10‐ to 50‐mg dose range, mean apomorphine C_max_ increased postdose and ranged from 4.16 to 9.29 ng/mL, with wide interpatient variability (coefficient of variation 18.6‒107%) and peak concentrations occurring from 0.5 to 1 hour postdose; increases in C_max_ with increases in dose were less than dose‐proportional (Figure 3). Systemic exposure, measured by AUC_last_, increased with an increase in dose after apomorphine sublingual film but was less than dose‐proportional. Moxifloxacin (400 mg) plasma concentration profiles had a C_max_ of 1920 ng/mL at 2 hours postdose, consistent with a previous report.^27^

### Safety

Most TEAEs occurred during the open‐label titration phase (41 patients [85.4%]) and were mild, self‐limiting, and considered treatment related. The most common (≥10%) TEAEs in the safety population (N = 48; Table 2) and in those who tolerated a higher dose level of apomorphine sublingual film than that which initially resulted in FULL “ON” (n = 35), respectively, were nausea (56.3% and 60.0%), somnolence (25.0% and 28.6%), vomiting (18.8% and 14.3%), dizziness (16.7% and 22.9%), and hyperhidrosis (14.6% and 11.4%). During the crossover phase, TEAEs were reported in 13 (32.5%), six (15.0%), and four (10.0%) patients after apomorphine sublingual film, placebo, and moxifloxacin administration, respectively. The most common (≥10%) TEAEs associated with apomorphine sublingual film were somnolence (15.0%) and nausea (10.0%; Table 2).

**Table 2:** TEAEs Reported by at Least Two Patients in any Treatment Group

	Open‐label Dose‐titration Phase (N = 48)	Randomized Crossover Phase (N = 40)		
TEAE	Apomorphine Sublingual Film, n (%)	Apomorphine Sublingual Film, n (%)	Moxifloxacin, n (%)	Placebo, n (%)
Any	41 (85.4)	13 (32.5)	4 (10.0)	6 (15.0)
Nausea	27 (56.3)	4 (10.0)	0	0
Somnolence	12 (25.0)	6 (15.0)	1 (2.5)	2 (5.0)
Vomiting	9 (18.8)	2 (5.0)	0	0
Dizziness	8 (16.7)	NR	NR	NR
Hyperhidrosis	7 (14.6)	1 (2.5)	0	0
Hypotension	4 (8.3)	1 (2.5)	0	0
Headache	3 (6.3)	NR	NR	NR
Orthostatic hypotension	3 (6.3)	1 (2.5)	2 (5.0)	0
Systolic blood pressure decreased	2 (4.2)	1 (2.5)	0	0
Dyskinesia	2 (4.2)	1 (2.5)	0	0
Vertigo	2 (4.2)	NR	NR	NR
Yawning	2 (4.2)	NR	NR	NR
Hypertension	NR	0	0	2 (5.0)

NR, not reported; TEAE, treatment‐emergent adverse event.

No TEAEs of syncope or hallucinations occurred with apomorphine sublingual film, and no suicidal ideation or attempts were reported.

Six TEAEs of severe intensity occurred during the study (five in the open‐label titration phase and one in the crossover phase), all in patients who received apomorphine sublingual film. Nausea was the most frequently reported severe TEAE (three patients, one event at 20 mg and two events at 10 mg) and led to study discontinuation in all cases. Severe somnolence (10 mg) and orthostatic hypotension (50 mg) occurred in one patient each and led to study discontinuation. Another patient experienced a >20 mmHg decrease in systolic blood pressure (10 mg) that did not lead to study discontinuation. No other patients with orthostatic hypotension and decreased systolic blood pressure were symptomatic. All events resolved. No serious TEAEs or deaths were reported.

## Discussion

This placebo‐ and moxifloxacin‐positive–controlled thorough QT study demonstrated that apomorphine sublingual film at 10‐ to 50‐mg doses did not have a clinically significant effect on the QTc interval in patients with PD. At each prespecified timepoint, the upper bound of the 90% CI for ΔΔQTcF did not exceed the regulatory threshold (10 milliseconds),^28^ showing that apomorphine sublingual film does not prolong the QT interval. However, it should be noted that the relatively small number of patients treated with higher doses of apomorphine sublingual film may limit the interpretation of the study results at these doses. In addition, this study does not exclude the possibility of prolongation in the presence of other treatments that can prolong the QT interval.

The validity of these results was supported by assay sensitivity in response to moxifloxacin versus placebo, for which the lower bound of the 90% CI exceeded the regulatory threshold (5 milliseconds) at three of four prespecified timepoints. Additionally, apomorphine sublingual film did not result in any deleterious effects on other cardiac conduction measures or ECG morphology as evidenced by comparable effects on cardiac parameters between apomorphine sublingual film and placebo.

In a pharmacokinetic comparative bioavailability study, apomorphine sublingual film had a lower C_max_ compared with subcutaneous apomorphine formulations at similar exposures.^18^ Importantly in the current study, QT prolongation was not observed at apomorphine C_max_, despite administration of apomorphine sublingual film doses exceeding those needed to achieve FULL “ON,” perhaps owing to the slower rate of absorption of sublingual versus subcutaneous apomorphine.^18^ There was a nonlinear relationship between C_max_ and dose, likely due to high interpatient variability. This relationship may have also been influenced by route of administration, which requires adequate moisture in the oral cavity to facilitate dissolution of the sublingual film. In addition, patients may have held the sublingual film under their tongue for different lengths of time. The mean minimal toxic range for apomorphine is 8.5‒16.7 ng/mL,^29^ and the range of median C_max_ observed at doses from 10 to 20 mg was 2.7‒4.1 ng/mL (range 0.8‒11.1 ng/mL) from several pooled apomorphine sublingual film studies (Agbo, unpublished data, 2019). Therefore, the typical C_max_ observed at lower doses of apomorphine sublingual film is much lower than the minimal toxic range. In general, AUC_last_ tended to increase over the 10‐ to 35‐mg dose range, but overall exposure (C_max_, AUC_last_, and AUC from time 0 to infinity) was less than dose proportional. The QT interval was independent of apomorphine concentrations across the range of apomorphine sublingual film doses evaluated in this study, including the highest approved dose of 30 mg.

The TEAEs observed with single doses of 10‒50 mg of apomorphine sublingual film in this study were consistent with those reported in the pivotal (12 weeks) and long‐term safety (48 weeks) studies.^19^, ^30^ Despite experiencing some TEAEs commonly associated with dopaminergic agents, such as nausea and somnolence, patients successfully titrated apomorphine sublingual film without the use of antiemetics, which were prohibited in this study. The higher rate of nausea compared with that previously reported reflected events that occurred during up‐titration to the second dose level after the initial FULL “ON” achieved at the lower dose level. In general, these findings suggest that antiemetics may not be needed for many patients during titration of apomorphine sublingual film.

Apomorphine sublingual film has demonstrated efficacy in improving motor function in patients with PD in a pivotal study.^19^ One patient in the pivotal study experienced a TEAE of QT prolongation at 35 mg.^19^ QT prolongation has been rarely reported in an ongoing long‐term safety and efficacy study of patients exposed to apomorphine sublingual film (n = 2/467 [0.4%], data on file). This stands in contrast with apomorphine administered as a subcutaneous injection, which is associated with dose‐related prolongation of the QTc interval at exposure to the higher end of the recommended dose range as described in the literature and approved product labeling.^16^

In conclusion, this placebo‐ and moxifloxacin‐positive–controlled thorough QT study in patients with PD and “OFF” episodes demonstrated that apomorphine sublingual film does not induce clinically significant effects on QT prolongation or other cardiac conduction parameters at the exposure levels evaluated. When administered at 10‐ to 50‐mg doses, TEAEs associated with apomorphine sublingual film were consistent with previous reports,^19^, ^30^ and no new or unexpected safety findings were identified. Overall, these data suggest that apomorphine sublingual film is not associated with QT prolongation for the approved dose range (10‒30 mg). As subcutaneous apomorphine is associated with an exposure‐related potential for QTc change, and the effect of sublingual apomorphine on QTc interval at higher doses remains somewhat uncertain, physicians should consider the risks and benefits of apomorphine sublingual film before initiating treatment in patients with risk factors for prolonged QTc.

## Conflicts of Interest

F.S. and E.L.P. received research/grant support from Sunovion Pharmaceuticals Inc. for participation in this study. M.F.D.P. reports no conflicts of interest. K.S. and F.A. are full‐time employees of Sunovion Pharmaceuticals Inc. and may hold stock/stock options in the company. R.K. is an employee of eResearch Technology Inc. (ERT) and performs consulting services for various pharmaceutical companies, including Sunovion Pharmaceuticals Inc., for which ERT receives payment. C.W.O. owns shares in Clintrex Research Corporation, which has received fees from Sunovion Pharmaceuticals Inc. D.B. and B.N. were employees of Sunovion Pharmaceuticals Inc. at the time the study was conducted.

## Funding

Supported by funding from Sunovion Pharmaceuticals Inc.

## Supporting information



Supporting information
