# Supplementary material for: A Randomized Thorough QT Study of Apomorphine Sublingual Film in Patients With Parkinson's Disease
Source: Clin Pharmacol Drug Dev. 2022 Jul 28;11(9):1068–77. doi: 10.1002/cpdd.1147 (PMC9541463; doi:10.1002/cpdd.1147)

**Supporting Information**

**Supporting Information File Legends**

**Table S1.** Highest apomorphine sublingual film dose received in the open-label dose-titration phase versus the randomized crossover phase

**Table S2.** Summary of change from baseline in QTcF (msec) by timepoint and treatment (ECG population)

**Table S3.** Number of patients with outliers in QTcF, QTcB, HR, and PR, QRS, and QT intervals by postdose timepoint and treatment group

**Table S4.** Summary of change from baseline in QRS interval (msec) by timepoint and treatment (ECG population)

**Table S5.** Summary of change from baseline in HR by timepoint and treatment (ECG population)

**Table S6.** Summary of change from baseline in PR interval (msec) by timepoint and treatment (ECG population)

**Table S7.** CTH-201 study investigators

**Figure S1.** Study design

**Figure S2.** Mean (SD) moxifloxacin concentration over time

**Figure S3.** Scatterplot of moxifloxacin concentration (ng/mL) versus moxifloxacin ΔΔQTcF (msec) with regression line

**Figure S4.** Scatterplot of RR interval (msec/beat) versus QTcF (msec) with regression line

**Table S1.** Highest apomorphine sublingual film dose received in the open-label dose-titration phase versus the randomized crossover phase

|  |  | Highest Apomorphine Sublingual Film Dose Received During Open-label Dose-Titration Phase | | | | | | | | | | |  |
| --- | --- | --- | --- | --- | --- | --- | --- | --- | --- | --- | --- | --- | --- |
| n (%)^a^ | Dose | | 10 mg | 15 mg | 20 mg | 25 mg | 30 mg | 35 mg | 40 mg | 50 mg | 60 mg | Total | |
| Highest apomorphine sublingual film dose received during crossover  phase | 10 mg | | 4 (100.0)^b^ | 11 (100.0) | 1 (5.9) | 0 | 0 | 0 | 0 | 0 | 0 | 16 | |
|  | 15 mg | | 0 | 0 | 3 (17.6) | 1 (20.0) | 0 | 0 | 0 | 0 | 0 | 4 | |
|  | 20 mg | | 0 | 0 | 13 (76.5) | 2 (40.0) | 0 | 0 | 0 | 0 | 0 | 15 | |
|  | 25 mg | | 0 | 0 | 0 | 2 (40.0) | 0 | 0 | 0 | 0 | 0 | 2 | |
|  | 30 mg | | 0 | 0 | 0 | 0 | 0 | 0 | 0 | 0 | 0 | 0 | |
|  | 35 mg | | 0 | 0 | 0 | 0 | 0 | 1 (100.0) | 2 (100.0) | 0 | 0 | 3 | |
|  | 40 mg | | 0 | 0 | 0 | 0 | 0 | 0 | 0 | 0 | 0 | 0 | |
|  | 50 mg | | 0 | 0 | 0 | 0 | 0 | 0 | 0 | 1 (100.0) | 0 | 1 | |
|  | 60 mg | | 0 | 0 | 0 | 0 | 0 | 0 | 0 | 0 | 0 | 0 | |
|  | Total | | 4 | 11 | 17 | 5 | 0 | 1 | 2 | 1 | 0 | 41 | |

^a^Percentages are based on the column total.

^b^Includes 1 patient who discontinued before dosing.

**Table S2.** Summary of change from baseline in QTcF (msec) by timepoint and treatment (ECG population)

| Timepoint | | | Value | Statistics | Apomorphine  Sublingual Film  (N=40) | Placebo  (N=40) | Moxifloxacin  (N=40) |
| --- | --- | --- | --- | --- | --- | --- | --- |
| Baseline^a^ | | | Observed | n | 40 | 40 | 40 |
|  | | |  | Mean (SD) | 407.5 (22.44) | 407.5 (22.44) | 407.5 (22.44) |
| 15 min postdose | | | CFB | n | 39 | 40 | 39 |
|  | | |  | Mean (SD) | 0.8 (9.37) | ‒3.1 (10.36) | ‒2.1 (10.70) |
| 30 min postdose | | | CFB | n | 39 | 40 | 39 |
|  | | |  | Mean (SD) | 0.9 (12.53) | ‒2.0 (8.71) | 1.1 (11.90) |
| 45 min postdose | | CFB | n | 39 | 40 | 39 |  |
|  | |  | Mean (SD) | 0.6 (9.77) | ‒3.0 (10.82) | 4.8 (11.03) |  |
| 1 hr postdose | | CFB | n | 39 | 40 | 39 |  |
|  | |  | Mean (SD) | 3.3 (9.20) | ‒2.9 (8.88) | 7.1 (11.65) |  |
| 2 hr postdose | | CFB | n | 39 | 40 | 38 |  |
|  | |  | Mean (SD) | 2.4 (11.60) | ‒2.4 (12.16) | 10.4 (11.60) |  |
| 3 hr postdose | CFB | | n | 39 | 39 | 39 |  |
|  |  | | Mean (SD) | 0.7 (14.28) | ‒0.9 (11.40) | 9.9 (13.76) |  |
| 4 hr postdose | CFB | | n | 39 | 39 | 39 |  |
|  |  | | Mean (SD) | ‒0.3 (14.62) | 0.5 (14.85) | 9.4 (14.12) |  |

CFB, change from baseline; ECG, electrocardiogram; QTcF, Fridericia-corrected QTc interval; SD, standard deviation.

^a^Baseline is predose of Period 1.

**Table S3.** Number of patients with outliers in QTcF, QTcB, HR, and PR, QRS, and QT intervals by postdose timepoint and treatment group^a^

| Time, hr | 0.25 | | | 0.5 | | | 0.75 | | | 1 | | | 2 | | | 3 | | | 4 | | | 8 | | | 12 | | | 24 | | |
| --- | --- | --- | --- | --- | --- | --- | --- | --- | --- | --- | --- | --- | --- | --- | --- | --- | --- | --- | --- | --- | --- | --- | --- | --- | --- | --- | --- | --- | --- | --- |
| Study drug | A | M | P | A | M | P | A | M | P | A | M | P | A | M | P | A | M | P | A | M | P | A | M | P | A | M | P | A | M | P |
| **QTcF** |  |  |  |  |  |  |  |  |  |  |  |  |  |  |  |  |  |  |  |  |  |  |  |  |  |  |  |  |  |  |
| >500 msec |  |  |  |  |  |  |  |  |  |  |  |  |  |  |  |  |  |  |  |  |  |  |  |  |  |  |  |  |  |  |
| >480 msec |  | 1 |  |  |  |  |  | 1 |  |  | 1 |  |  | 1 |  |  | 1 |  |  |  |  |  |  |  |  | 1 |  |  |  |  |
| >450 msec |  | 1 |  |  | 1 |  | 1 |  |  | 2 | 2 |  | 3 | 2 | 1 | 1 | 4 | 1 | 1 | 4 | 2 | 1 | 1 |  |  | 2 |  | 2 | 1 | 2 |
| >30- to 60-msec increase |  |  |  |  |  |  |  |  | 1 |  | 1 |  |  | 2 | 1 |  | 2 |  | 2 | 2 | 1 | 1 |  |  |  | 4 |  |  | 1 |  |
| >60-msec increase |  |  |  |  |  |  |  |  |  |  |  |  |  |  |  |  |  |  |  |  |  |  |  |  |  |  |  |  |  |  |
| **QTcB** |  |  |  |  |  |  |  |  |  |  |  |  |  |  |  |  |  |  |  |  |  |  |  |  |  |  |  |  |  |  |
| >500 msec |  |  |  |  |  |  |  |  |  |  |  |  |  |  |  |  |  |  |  |  |  |  |  |  |  |  |  |  |  |  |
| >480 msec |  |  |  |  |  |  |  | 1 |  |  | 1 |  |  | 1 |  |  | 1 |  | 1 | 2 |  | 1 |  |  | 1 | 1 |  |  |  |  |
| >450 msec | 2 | 1 |  | 1 |  |  | 3 | 2 | 1 | 2 | 3 |  | 3 | 2 | 3 | 4 | 3 | 1 | 5 | 5 | 4 | 3 | 2 | 2 | 1 | 2 | 1 | 2 | 2 | 2 |
| >30- to 60-msec increase | 2 | 1 |  |  | 1 |  |  | 2 | 1 |  | 1 | 1 | 1 | 3 | 1 | 4 | 2 | 1 | 4 | 3 | 3 | 1 | 1 | 1 | 2 | 3 | 1 | 1 | 3 | 1 |
| >60-msec increase |  |  |  |  |  |  |  |  |  |  |  |  |  |  |  |  |  |  |  | 1 |  |  |  |  |  |  |  |  |  |  |
| **HR** |  |  |  |  |  |  |  |  |  |  |  |  |  |  |  |  |  |  |  |  |  |  |  |  |  |  |  |  |  |  |
| Bradycardic event |  |  |  |  |  |  |  |  |  |  |  |  |  |  |  |  |  |  |  |  |  |  |  |  |  |  |  |  |  |  |
| Tachycardic event |  |  |  |  |  |  |  |  | 1 |  | 2 |  | 1 |  | 1 |  |  |  | 2 |  | 1 | 3 | 2 | 1 |  | 1 | 1 | 1 |  | 3 |
| Bradycardic or tachycardic event |  |  |  |  |  |  |  |  | 1 |  | 2 |  | 1 |  | 1 |  |  |  | 2 |  | 1 | 3 | 2 | 1 |  | 1 | 1 | 1 |  | 3 |
| **PR** >200 msec and increase ≥25% |  |  |  |  |  |  |  |  |  |  |  |  |  |  |  |  |  |  |  |  |  |  |  |  |  |  |  |  |  |  |
| **QRS** >100 msec and increase ≥25% |  |  |  |  |  |  |  |  |  |  |  |  | 1 |  |  | 1 |  | 1 | 1 | 1 | 1 | 1 |  | 1 |  |  |  |  |  |  |
| **QT** >500 msec |  |  |  |  |  |  |  |  |  |  |  |  | 1 |  |  | 1 |  |  |  |  |  |  |  |  |  |  |  |  |  |  |

A, apomorphine sublingual film; ECG, electrocardiogram; HR, heart rate; M, moxifloxacin; P, placebo; QTcB, Bazett-corrected QTc interval; QTcF, Fridericia-corrected QTc interval.

^a^N=40 (ECG population).

**Table S4.** Summary of change from baseline in QRS interval (msec) by timepoint and treatment (ECG population)

| Timepoint | Value | Statistics | Apomorphine  Sublingual Film  (N=40) | Placebo  (N=40) | Moxifloxacin  (N=40) |
| --- | --- | --- | --- | --- | --- |
| Baseline^a^ | Observed | n | 40 | 40 | 40 |
|  |  | Mean (SD) | 91.3 (7.56) | 91.3 (7.56) | 91.3 (7.56) |
| 15 min postdose | CFB | n | 39 | 40 | 39 |
|  |  | Mean (SD) | ‒0.5 (4.39) | ‒1.0 (4.38) | ‒0.6 (4.64) |
| 30 min postdose | CFB | n | 39 | 40 | 39 |
|  |  | Mean (SD) | ‒0.2 (4.54) | ‒0.7 (4.10) | 0.0 (4.39) |
| 45 min postdose | CFB | n | 39 | 40 | 39 |
|  |  | Mean (SD) | ‒1.0 (4.56) | 0.2 (6.41) | ‒0.7 (3.84) |
| 1 hr postdose | CFB | n | 39 | 40 | 39 |
|  |  | Mean (SD) | ‒0.6 (3.84) | ‒1.1 (4.52) | ‒0.2 (4.18) |
| 2 hr postdose | CFB | n | 39 | 40 | 38 |
|  |  | Mean (SD) | 1.3 (6.63) | ‒1.0 (5.51) | ‒1.0 (4.48) |
| 3 hr postdose | CFB | n | 39 | 39 | 39 |
|  |  | Mean (SD) | 0.0 (6.95) | ‒0.5 (7.03) | ‒0.8 (4.41) |
| 4 hr postdose | CFB | n | 39 | 39 | 39 |
|  |  | Mean (SD) | 0.7 (6.26) | ‒0.3 (7.72) | 0.6 (6.72) |

CFB, change from baseline; ECG, electrocardiogram; SD, standard deviation.

^a^Baseline is predose of Period 1.

**Table S5.** Summary of change from baseline in HR by timepoint and treatment (ECG population)

| Timepoint | | Value | | Statistics | | Apomorphine Sublingual Film  (N=40) | Placebo  (N=40) | Moxifloxacin  (N=40) |
| --- | --- | --- | --- | --- | --- | --- | --- | --- |
| Baseline^a^ | | Observed | | n | | 40 | 40 | 40 |
|  | |  | | Mean (SD) | | 72.4 (11.46) | 72.4 (11.46) | 72.4 (11.46) |
| 15 min postdose | | CFB | | n | | 39 | 40 | 39 |
|  | |  | | Mean (SD) | | 1.9 (7.19) | 1.2 (7.19) | ‒0.6 (8.38) |
| 30 min postdose | | CFB | | n | | 39 | 40 | 39 |
|  | |  | | Mean (SD) | | 0.8 (5.96) | 1.3 (8.11) | ‒0.2 (8.41) |
| 45 min postdose | | CFB | | n | 39 | 40 | 39 |  |
|  | |  | | Mean (SD) | ‒0.5 (8.37) | ‒0.1 (9.96) | 2.8 (8.02) |  |
| 1 hr postdose | | CFB | | n | 39 | 40 | 39 |  |
|  | |  | | Mean (SD) | ‒3.7 (7.58) | ‒0.7 (8.75) | 1.1 (10.03) |  |
| 2 hr postdose | | CFB | | n | 39 | 40 | 38 |  |
|  | |  | | Mean (SD) | 2.7 (9.73) | 2.0 (8.72) | 0.8 (7.76) |  |
| 3 hr postdose | | CFB | | n | 39 | 39 | 39 |  |
|  | |  | | Mean (SD) | 6.9 (9.11) | 3.8 (8.42) | 2.5 (6.71) |  |
| 4 hr postdose | | CFB | | n | 39 | 39 | 39 |  |
|  | |  | | Mean (SD) | 8.9 (9.21) | 5.4 (9.57) | 2.2 (6.75) |  |

CFB, change from baseline; ECG, electrocardiogram; HR, heart rate; SD, standard deviation.

^a^Baseline is predose of Period 1.

**Table S6.** Summary of change from baseline in PR interval (msec) by timepoint and treatment (ECG population)

| Timepoint | | | Value | Statistics | Apomorphine  Sublingual Film  (N=40) | Placebo  (N=40) | | Moxifloxacin  (N=40) | |
| --- | --- | --- | --- | --- | --- | --- | --- | --- | --- |
| Baseline^a^ | | | Observed | n | 40 | 40 | | 40 | |
|  | | |  | Mean (SD) | 158.2 (17.46) | 158.2 (17.46) | | 158.2 (17.46) | |
| 15 min postdose | | | CFB | n | 39 | 40 | | 39 | |
|  | | |  | Mean (SD) | 0.9 (11.21) | ‒1.1 (11.07) | | ‒0.3 (9.82) | |
| 30 min postdose | | | CFB | n | 38 | 40 | | 39 | |
|  | | |  | Mean (SD) | ‒1.0 (13.16) | 0.5 (9.43) | | 0.6 (9.72) | |
| 45 min postdose | | CFB | n | 39 | | 40 | | 39 | |
|  | |  | Mean (SD) | 1.7 (10.47) | | 0.8 (11.42) | | ‒0.2 (11.39) | |
| 1 hr postdose | | CFB | n | 39 | | 40 | | 39 | |
|  | |  | Mean (SD) | 3.1 (9.08) | | 0.5 (9.44) | | 2.4 (11.34) | |
| 2 hr postdose | | CFB | n | 39 | | 40 | | 38 | |
|  | |  | Mean (SD) | 0.3 (11.90) | | ‒1.0 (12.68) | | 1.3 (9.34) | |
| 3 hr postdose | CFB | | n | 38 | | 39 | | 39 | |
|  |  | | Mean (SD) | ‒1.0 (10.12) | | ‒1.4 (11.29) | | 2.4 (11.64) | |
| 4 hr postdose | CFB | | n | 38 | | 39 | | 39 | |
|  |  | | Mean (SD) | ‒3.2 (12.73) | | ‒2.2 (9.56) | | 1.6 (10.41) | |

CFB, change from baseline; ECG, electrocardiogram; SD, standard deviation.

^a^Baseline is predose of Period 1.

**Table S7.** CTH-201 study investigators

| Site No. | Principal Investigator | Institution | Location | Country |
| --- | --- | --- | --- | --- |
| 4001 | Fabrizio Stocchi | IRCCS San Raffaele Pisana | Via della Pisana, 235  00163 Rome | Italy |
| 4002 | Alessandro Stefani | Policlinico Tor Vergata | Viale Oxford, 81  00133 Rome | Italy |
| 4003 | Maria Francesca De Pandis | IRCCS San Raffaele Cassino | via G. Di Biasio, 1  03043 Cassino (FR) | Italy |
| 4004 | Marco Onofrj | Aging Research Center Ce.S.I.  University Foundation, Chieti-Pescara  Behavioural Neurology and Movement Disorders Unit | Via Luigi Polacchi, 11  66100 Chieti | Italy |
| 4101 | Kerri Wilks | Wilks & Safirstein MD PA D/B/A MD Clinical | 911 E Hallandale Beach Blvd, Hallandale Beach, FL 33009-4427 | USA |
| 4103 | Ira Goodman | Bioclinica Research | 100 West Gore St, Suite 202, Orlando, FL 32806 | USA |
| 4104 | Victor Biton | Clinical Trials Inc. | Marketing, Bldg, 2 Lile Ct, Ste 100, Little Rock, AR 72205-6241 | USA |
| 4105 | Robert Riesenberg | Atlanta Center for Medical Research | 501 Fairburn Rd Sw, Atlanta, GA 30331-2012 | USA |
| 4106 | Elizabeth Peckham | Central Texas Neurology Consultants | 16040 Park Valley Dr, Ste B-100, Round Rock, TX 78681-3579 | USA |
| 4107 | Daniel Truong | The Parkinsons and Movement Disorder Institute | 9940 Talbert Ave, Ste 204, Fountain Valley, CA 92708-5153 | USA |
| 4108 | Stuart Isaacson | Parkinsons Disease and Movement Disorders Center of Boca Raton | 951 NW 13th St, Bldg 5E,  Boca Raton, FL 33486-2359 | USA |
| 4109 | Ivan Bodis-Wollner | SUNY Downstate Medical Center, Department of Neurology | 450 Clarkson Ave, MSC 1213, Brooklyn, NY 11203-2012 | USA |
| 4111 | Virgilio Evidente | Movement Disorders Center of Arizona | 9590 E Ironwood Square Dr, MD, Scottsdale, AZ 85258-4581 | USA |

**Figure S1.** Study design. ^a^During the open-label titration phase, dose increases occurred on subsequent days during the first 2 titration visits. During titration visits 3‒6, patients could receive the next highest dose after 4 hours if an “OFF” episode occurred that day, with a maximum of 2 doses per day. Dosing returned to 1 dose per day for titration visits 7‒9.


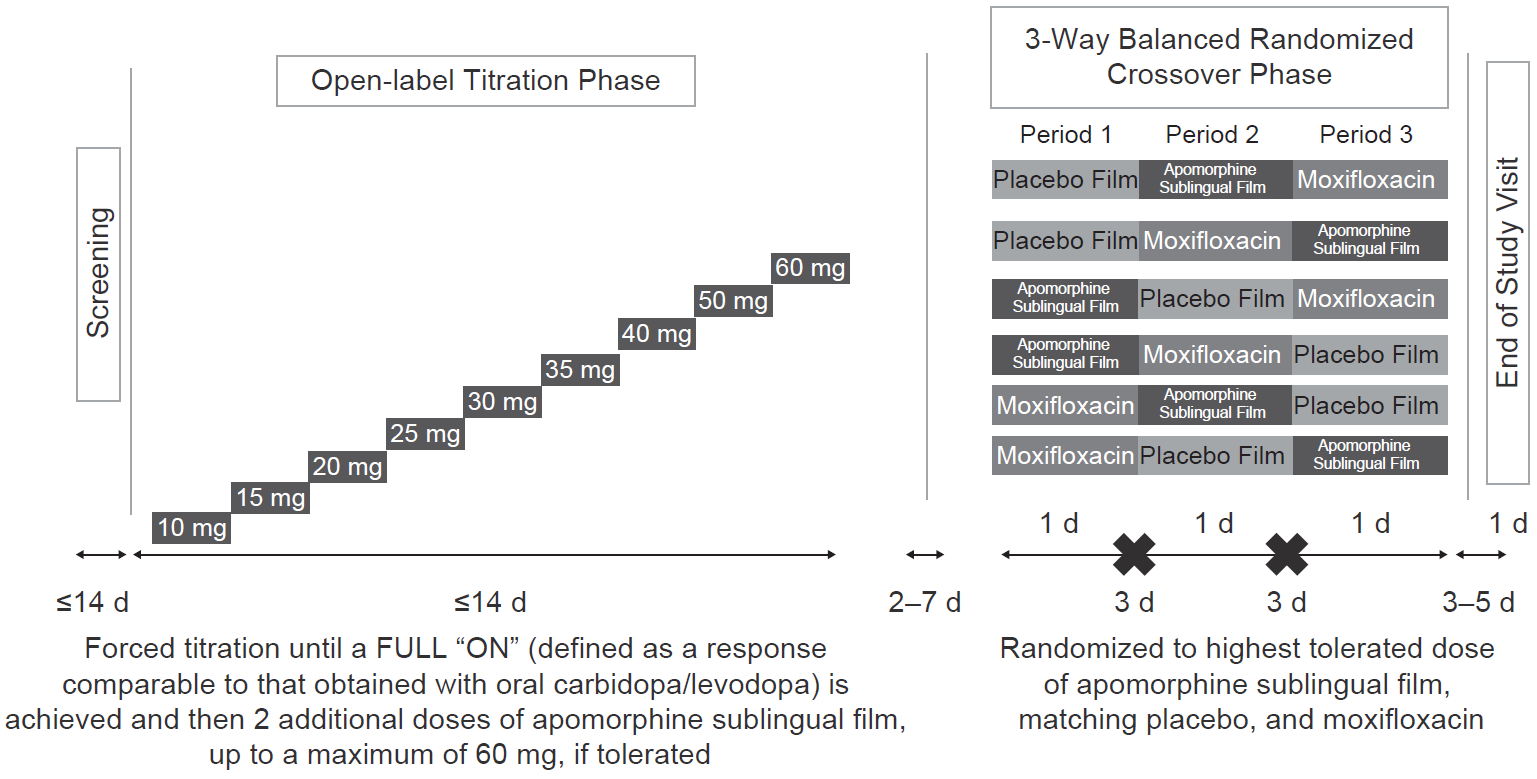


**Figure S2.** Mean (SD) moxifloxacin concentration over time. SD, standard deviation.


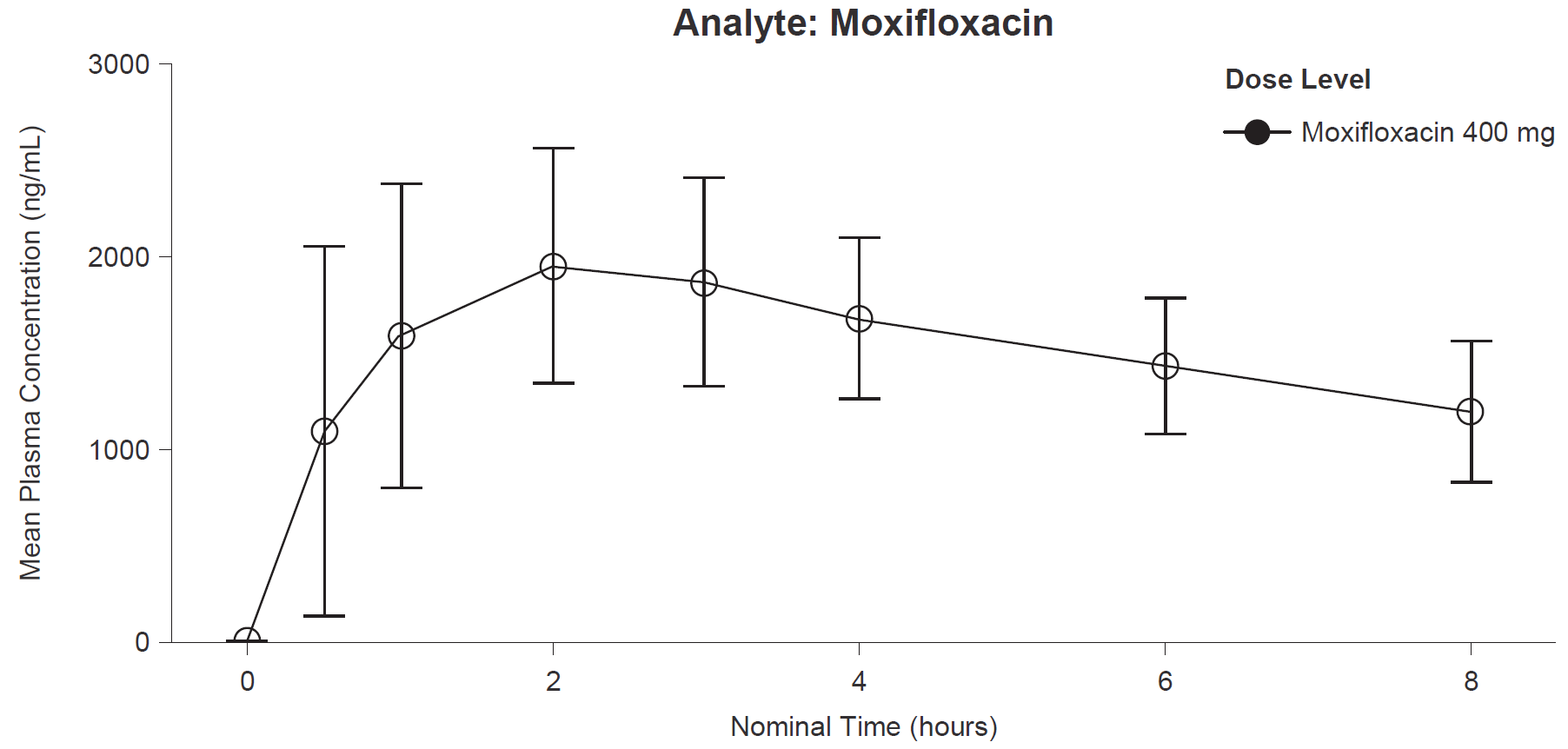


**Figure S3.** Scatterplot of moxifloxacin concentration (ng/mL) versus moxifloxacin ΔΔQTcF (msec) with regression line. ΔΔQTcF, baseline- and placebo-normalized Fridericia-corrected QTc interval.


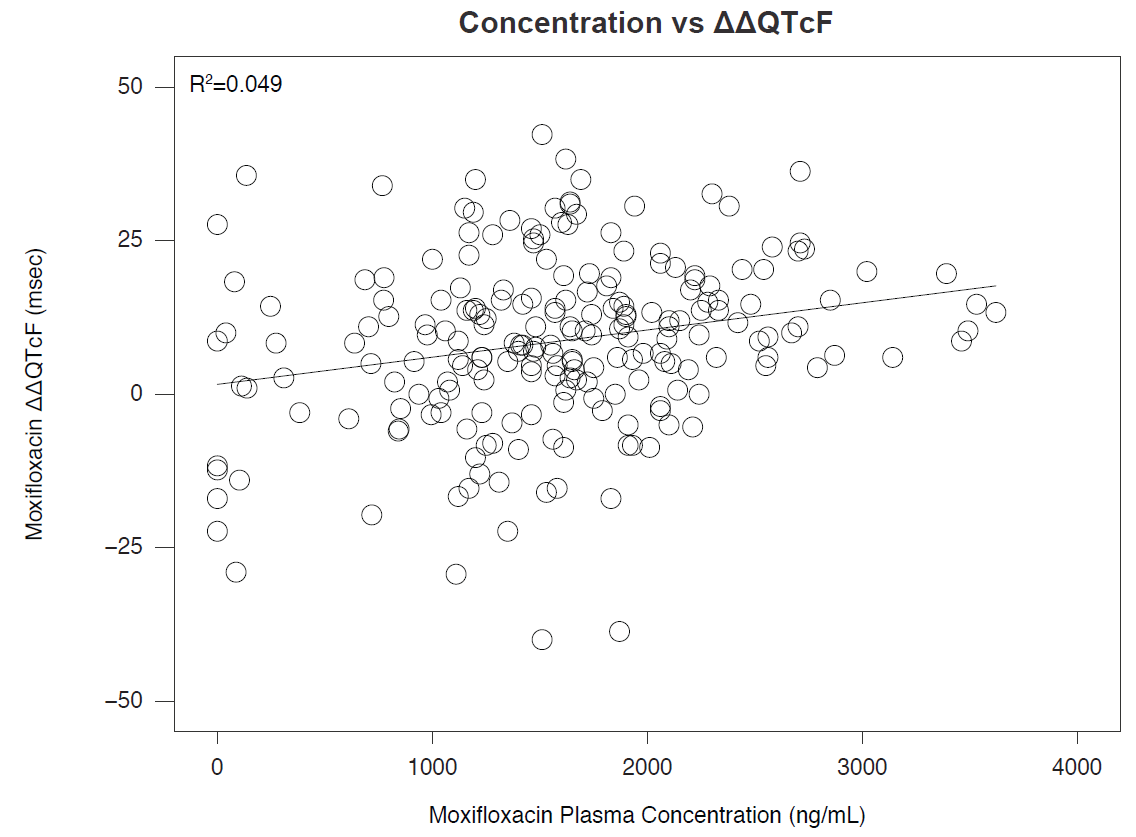


**Figure S4.** Scatterplot of RR interval (msec/beat) versus QTcF (msec) with regression line. QTcF, Fridericia-corrected QTc interval.


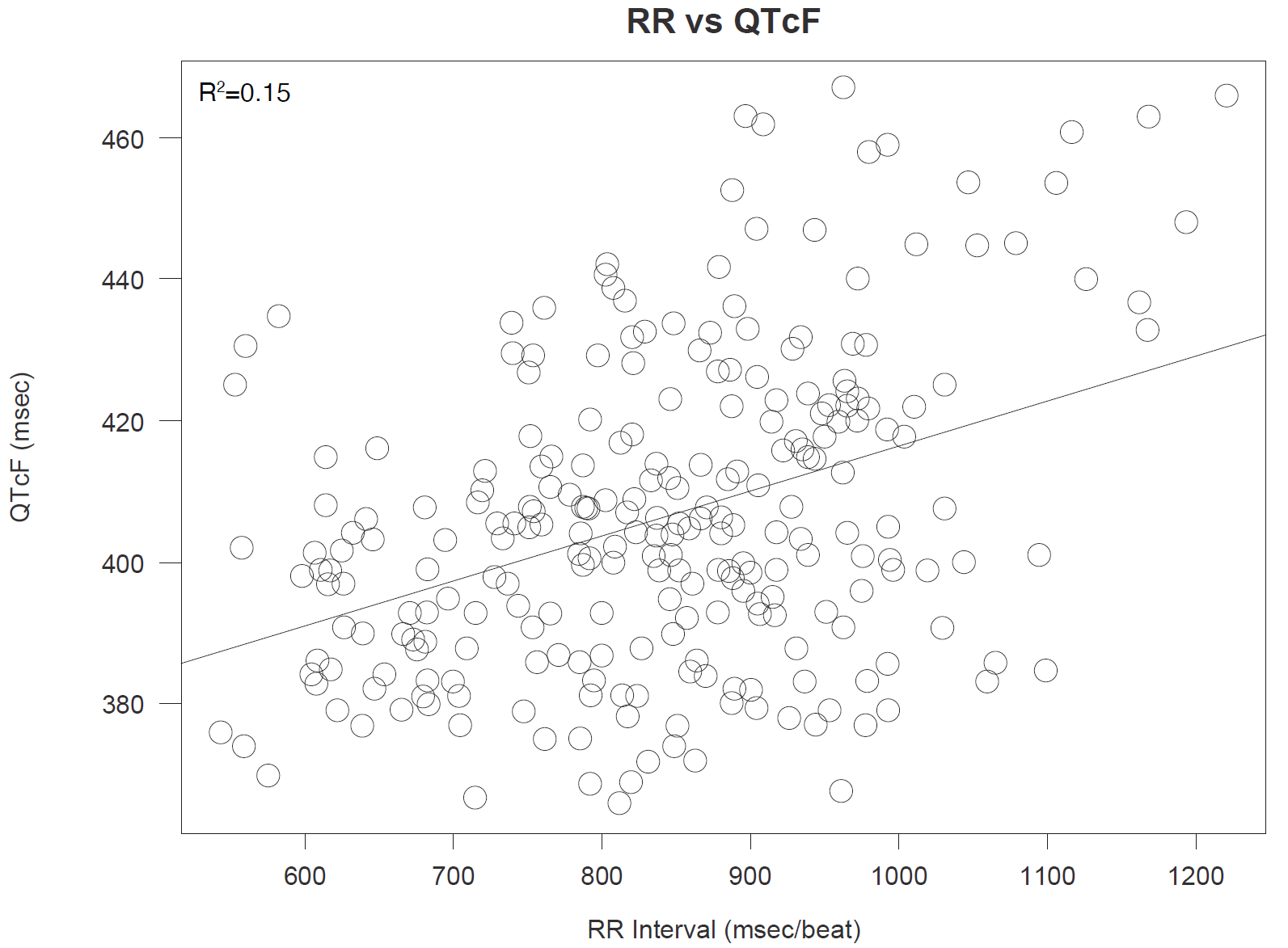

Supplement: Supplementary file 1 — Supporting information [file CPDD-11-1068-s001.docx]
